# CD4-mimetics sensitize HIV-infected cells to ADCC mediated by plasma from persons with early-stage HIV-1 infection

**DOI:** 10.1128/jvi.00858-25

**Published:** 2025-07-21

**Authors:** Shilei Ding, Alexandra Tauzin, Delia Pinto-Santini, Sayan Dasgupta, Derek Yang, William D. Tolbert, Monika Chandravanshi, Mehdi Benlarbi, Myriam Verly, Javier R. Lama, Donna M. Huryn, Amos B. Smith, Marzena Pazgier, Andrés Finzi, Ann Duerr

**Affiliations:** 1 Centre de Recherche du CHUM 177460, Montreal, Québec, Canada; 2 Département de Microbiologie, Infectiologie et Immunologie, Université de Montréal 5622, Montreal, Québec, Canada; 3 Vaccine and Infectious Disease Division, Fred Hutchinson Cancer Center 7286, Seattle, Washington, USA; 4 Department of Chemistry, School of Arts and Sciences, University of Pennsylvania 142839 , Philadelphia, Pennsylvania, USA; 5 Infectious Disease Division, Department of Medicine, Uniformed Services University of the Health Sciences 1685, Bethesda, Maryland, USA; 6 Asociación Civil Impacta Salud y Educación 585986, Lima, Peru; Icahn School of Medicine at Mount Sinai, New York, New York, USA

**Keywords:** HIV-1, plasma from PLWH, ADCC, CD4-mimetic compounds

## Abstract

The viral reservoir in long-lived memory CD4+ cells, established in the early stages of HIV infection, represents the main obstacle to an HIV cure. Some strategies being developed to target the reservoir rely on rendering HIV-1 envelope glycoproteins (Env) visible to the immune system. Small molecule CD4-mimetics (CD4mcs) expose vulnerable Env epitopes, which can be targeted by non-neutralizing antibodies (nnAbs) that are abundant in the plasma of people living with HIV (PLWH) and can mediate antibody-dependent cellular cytotoxicity (ADCC). Administration of CD4mcs in combination with plasma from PLWH or nnAbs efficiently reduces the size of the HIV-1 reservoir and postpones viral rebound upon antiretroviral therapy (ART) interruption in humanized mice. However, it remains unclear when these nnAbs are elicited after HIV infection. In this study, we collected longitudinal plasma samples before acquisition, at diagnosis, and at multiple time points up to 33 weeks after the estimated date of detectable infection (EDDI) and before ART treatment initiation. We found that plasma samples collected as early as 3 to 10 weeks after EDDI neutralized viral particles and mediated ADCC in the presence of the CJF-III-288 CD4mc. Recognition of HIV-1-infected cells and ADCC progressively increased over time, reaching a plateau by 19–25 weeks after EDDI. ADCC activity increased concomitantly with the elicitation of anti-gp120 and anti-gp41 CD4-induced (CD4i) Abs and improved over time with the appearance of anti-coreceptor binding site antibodies. Our results show that CD4i nnAbs, able to eliminate HIV-1-infected cells in the presence of CD4mc, are elicited within a few weeks after HIV acquisition.

**IMPORTANCE:**

A viral reservoir is established at the early stages of HIV-1 infection. This reservoir persists during antiretroviral therapy (ART) treatment, and viral rebound is observed after ART interruption. New strategies are needed to reduce the size of the viral reservoir and prevent virus rebound. Several families of non-neutralizing antibodies, which are abundant in plasma from people living with HIV-1, neutralize viral particles and mediate the elimination of HIV-1-infected cells through antibody-dependent cellular cytotoxicity (ADCC) when combined with CD4-mimetic compounds. The presence of non-neutralizing antibodies in plasma during early-stage HIV-1 infection would support the use of CD4-mimetic compounds as an early intervention to decrease the size of the latent HIV-1 reservoir by eliminating infected cells.

## INTRODUCTION

HIV-1 establishes a persistent reservoir at the very early stages of infection (1, 2). While antiretroviral therapy (ART) reduces viral load to low or undetectable levels (3–8), it does not prevent or eradicate the viral reservoir, which is composed notably of long-lived memory CD4+ T cells (9, 10). As a result, the virus rebounds within weeks after ART interruption. New strategies specifically aimed at targeting the reservoir, the source of the rebound virus, are needed.

Antibody-dependent cellular cytotoxicity (ADCC) has been reported to be effective at eliminating HIV-1-infected cells *in vitro*, *ex vivo,* and *in vivo* (11–19). In the vast majority of people living with HIV (PLWH), HIV-1 infection elicits non-neutralizing antibodies directed against CD4-induced (CD4i) epitopes (20). These antibodies are non-neutralizing due to their inability to recognize the unliganded HIV-1 envelope glycoprotein (Env), explaining their inability to block infection by viral particles. The CD4i nature of the epitopes that these antibodies recognize also precludes them from mediating ADCC against productively infected cells. This is explained by the activities of Nef and Vpu, two accessory proteins used by HIV-1 to downregulate CD4, thereby preventing interactions of CD4 with Env, which would otherwise “open-up” Env, exposing CD4i epitopes and sensitizing infected cells to ADCC (12, 13).

Small molecule CD4-mimetic (CD4mc) compounds bind to the Phe43 cavity of gp120 and induce conformational changes in Env, resulting in the exposure of epitopes recognized by CD4i Abs (21, 22). These antibodies, which are abundant in the plasma of PLWH, can then eliminate infected cells by ADCC (20, 23–28). Of note, treatment of humanized mice (hu-mice) with a cocktail of a CD4mc and two CD4i Abs, one targeting the gp120-inner domain cluster A region and another targeting the coreceptor binding site (CoRBS), resulted in a significant decrease in the size of the viral reservoir and delayed time to viral rebound (29). However, it remains unclear when these Abs are elicited in the early stages of HIV-1 infection. If they are rapidly elicited, then CD4mc has the potential to be used shortly after HIV acquisition to prevent or decrease the viral reservoir.

Here, we analyzed longitudinal plasma samples collected before acquisition, at diagnosis, and at multiple time points after HIV acquisition from participants in the Sabes study, an acute HIV infection cohort in Lima, Peru (30). We evaluated samples from 17 participants collected at multiple time points shortly after HIV acquisition, as well as pre-infection samples from a subset, to assess the kinetics of the development of CD4i Abs capable of eliminating productively HIV-1-infected cells in the presence of a potent CD4mc.

## RESULTS

### Plasma sample collection from the early stage of HIV-1 infection

Samples included in this study were from 17 participants in the Sabes study, a study conducted in Lima, Peru, which was designed to prevent onward transmission of HIV by identifying HIV-negative, high-risk individuals, testing them monthly for HIV, and then rapidly treating those who acquired HIV. From July 2013 to September 2015, we screened 3,349 potential participants for HIV; most participants were men who have sex with men (MSM) or transgender women—the two groups at highest risk for HIV in Peru. A total of 2,682 (80.4%) were uninfected, and 2,109 began follow-up for 2 years with monthly HIV testing by serology and HIV RNA. We identified 256 incident infections (incidence: 10.4/100 person-years) (30). A total of 216 participants were diagnosed with acute (HIV RNA+/seronegative) or recent (seropositive with a negative test within 3 months) HIV-1 infection. These participants were rapidly linked to the study clinics, enrolled in the treatment phase of the study, and followed for up to 10 years. Blood samples were collected monthly prior to HIV acquisition, at the first visit after HIV diagnosis (“enrollment” or week 0), and at frequent time points thereafter, including 8, 16, and 24 weeks after enrollment. The time of HIV acquisition (estimated date of detectable infection [EDDI]) was estimated using a published algorithm based on the timing, results, and uncertainty windows of all HIV tests (31, 32). Participants with incident HIV were enrolled in the Sabes study (30) prior to local adoption of the WHO “Treat All” guidelines. At enrollment, participants were randomized to begin ART immediately or to defer until 24 weeks later; participants were not assigned to the deferred arm or, if assigned, were initiated on ART if they met local criteria (based on CD4 thresholds or WHO clinical staging) or if treatment was indicated in the judgment of the study clinician. Thus, participants started ART at or before they met thresholds set by local guidelines; such a study of randomized time of ART initiation could not be conducted now, in the era of universal treatment. The study was approved by ethics review boards at the Fred Hutchinson Cancer Center (Seattle) and the IMPACTA NGO (Lima); all participants gave informed consent for study participation and use of samples for future research.

Participants were young (median age 23 years for all participants, 28 years for the subset whose pre-infection samples were analyzed) with high viral load (VL), as expected in early, untreated HIV infection. They identified either as MSM (cisgender male) or transgender women (Table 1). All participants included in this analysis had been randomized to begin ART 24 weeks after enrollment (33). Plasma samples from 17 participants collected at four visits (weeks 0, 8, 16, 24) before ART initiation were used for the present study (Table 2). Plasma samples collected before HIV-1 acquisition from 5 of the 17 participants were used as controls.

**TABLE 1:** Demographics of Sabes study participants included in analysis

Characteristic	Value
Gender identity, no. (%)	
Cisgender male	15 (88%)
Transgender female	2 (12%)
Education, no. (%)	
Primary	1 (6%)
Secondary	5 (29%)
Post-secondary	11 (65%)
Below minimum wage, no. (%)^*a*^	8 (48%)
Age (yr) at diagnosis, median (range)	23 (18–41)
Log VL (Log copies/mL) at enrollment, median (range)	6.2 (3.9–7.1)
CD4 count (cells/mm³) at enrollment, median (range)	403 (148–831)
CD8 count (cells/mm³) at enrollment, median (range)	849 (298–1,474)

^*a*^ Minimum monthly wage at the time was 750 Nuevos Soles (~250 US dollars).

**TABLE 2:** Description of specimens analyzed

Group	*N* (samples)	Weeks from EDDI^*a*^	Weeks from enrollment	Weeks from ART	Viral load^*a*^ (Log copies/mL)	CD4 count^*a*^ (cells/mm^3^)
Pre-infection control	5	−8 (−17 to −2)	−26 to −9	−50 to −33	NA^*b*^	NA
1	17	5.0 (3–10)	0	−24	6.2 (3.9–7.1)	403 (148–831)
2	17	14.4 (11–17)	8	−16	NA	NA
3	17	21.0 (19–25)	16	−8	NA	NA
4	17	29.3 (27–33)	24	0	5.1 (1.8–6.0)	395 (98–736)

^*a*^ Values displayed are medians, with ranges in parentheses.

^*b*^ NA indicates not available.

### A small molecule CD4-mimetic sensitizes HIV-1-infected cells to ADCC mediated by antibodies elicited during early-stage HIV-1 infection

**Fig 1:**
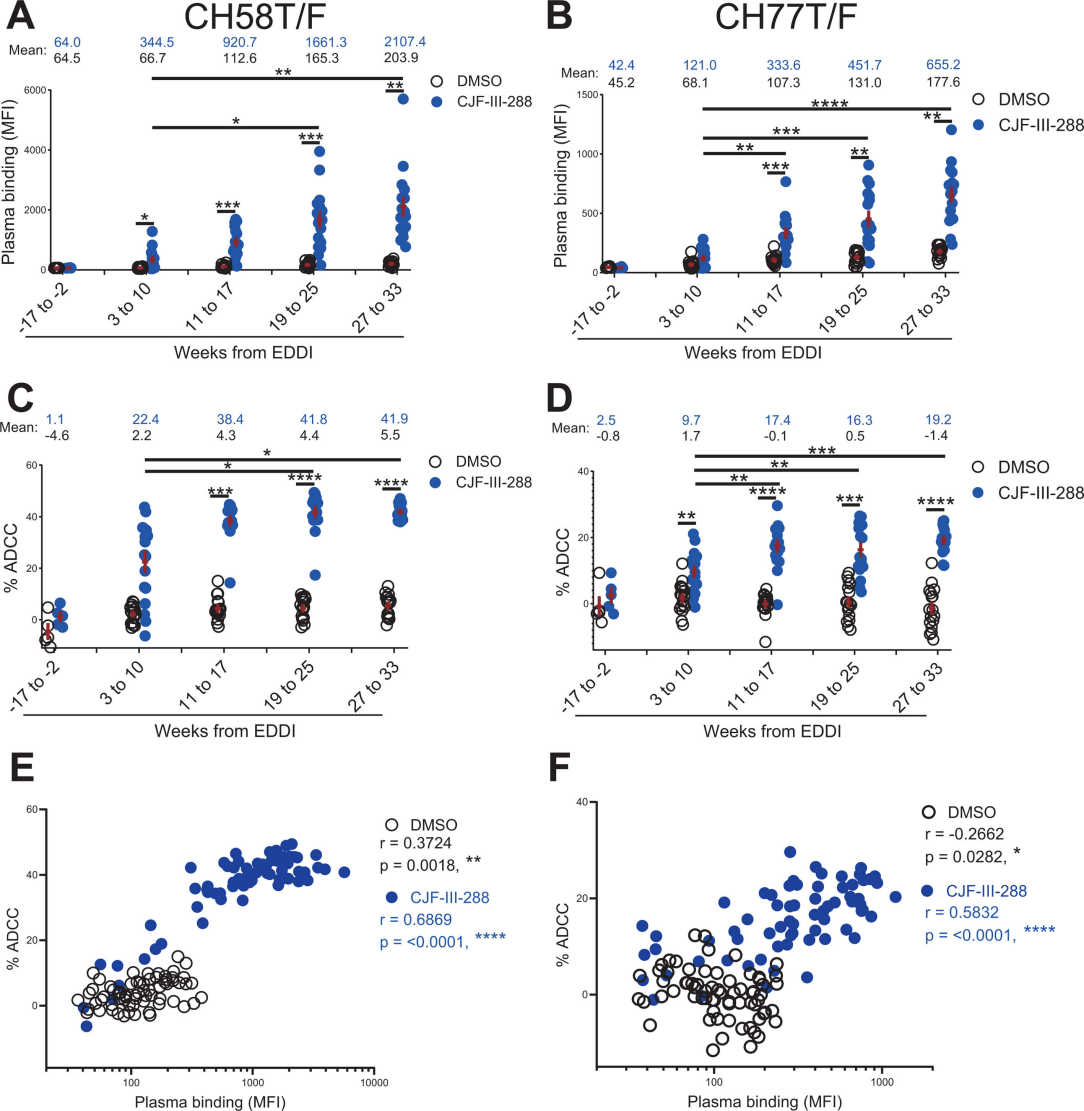
CD4-mimetics (CD4mcs) sensitize HIV-1-infected cells to ADCC mediated by plasma from early-stage PLWH. Primary CD4+ T cells isolated from resting peripheral blood mononuclear cells (PBMCs) were infected with HIV-1_CH58T/F_ (**A, C, and E**) or HIV-1_CH77T/F_ (**B, D, and F**) viruses for 48 h before performing staining and ADCC. (A–B) Surface staining of HIV-1-infected cells was done with a 1:1,000 dilution of plasma from PLWH. The samples were separated into five different groups according to their weeks from EDDI. Plasma was added in the presence of 5 µM of CJF-III-288 (blue filled circle) or an equivalent volume of the vehicle DMSO (black clear circle). The median fluorescence intensity (MFI) of secondary Ab (Alexa Fluor 647) is shown. (C–D) For ADCC, infected primary CD4+ T cells (target cells) were incubated with KHYG-1 cells (effector cells, ratio effector: target = 10:1) in the presence of a 1:1,000 dilution of plasma and 5 µM of CJF-III-288 (blue filled circle) or an equivalent volume of DMSO (black clear circle). The percentage of ADCC killing is shown. For A to D, statistical significance was tested with the Friedman test (*P < 0.05; **P < 0.01; ***P < 0.001; ****P < 0.0001). (**E and F**) Spearman correlations between staining (MFI) and ADCC in the presence of 5 µM of CJF-III-288 (blue filled circle) or an equivalent volume of DMSO (black clear circle) were calculated (*P < 0.05; **P < 0.01; ***P < 0.001; ****P < 0.0001).

Plasma samples were divided into five groups, according to time since EDDI (Table 2, Fig. 1A through 1D). To assess the ability of plasma to recognize and kill infected cells by ADCC, HIV-1_CH58T/F_ (Fig. 1A, C, and E) or HIV-1_CH77T/F_ (Fig. 1B, D, and F) infected primary CD4+ T cells were incubated with plasma samples (1/1,000 dilution) alone or in combination with 5 µM of an indoline CD4mc—CJF-III-288 (25). As expected, productively HIV-1-infected cells (CD4low/p24+) were not detected by plasma collected before HIV-1 infection (Fig. 1A and B) and therefore did not mediate ADCC in the absence or presence of CJF-III-288 (Fig. 1C and D). Interestingly, plasma samples collected early after HIV-1 infection (3–10 weeks post-EDDI) recognized HIV-1-infected cells upon addition of CJF-III-288 (Fig. 1A and B), and this recognition led to ADCC, effectively eliminating productively infected cells (Fig. 1C and D). Of note, despite the relatively low-level recognition of infected cells by plasma collected in weeks 3–10 post-EDDI compared to samples collected after 19 weeks, ADCC activity increased significantly in the presence of CJF-III-288, suggesting that antibodies elicited in early HIV-1 infection synergize effectively with CD4mc to eliminate HIV-1-infected cells.

With increased time post-EDDI, the level of antibodies in the plasma increased, as seen in the rise in both staining and ADCC activity against HIV-1-infected cells in the presence of CJF-III-288. The level of ADCC reached a plateau after week 19 post-EDDI in both HIV-1_CH58T/F_ and HIV-1_CH77T/F_ infected cells, though the staining increased steadily from week 19 to week 27 post-EDDI. Correlation analysis between staining and ADCC (Fig. 1E and F) indicates that higher plasma binding to HIV-1-infected cells correlates with increased ADCC activity in the presence of CJF-III-288.

### Kinetics of CD4i Abs elicitation in plasma from PLWH

**Fig 2:**
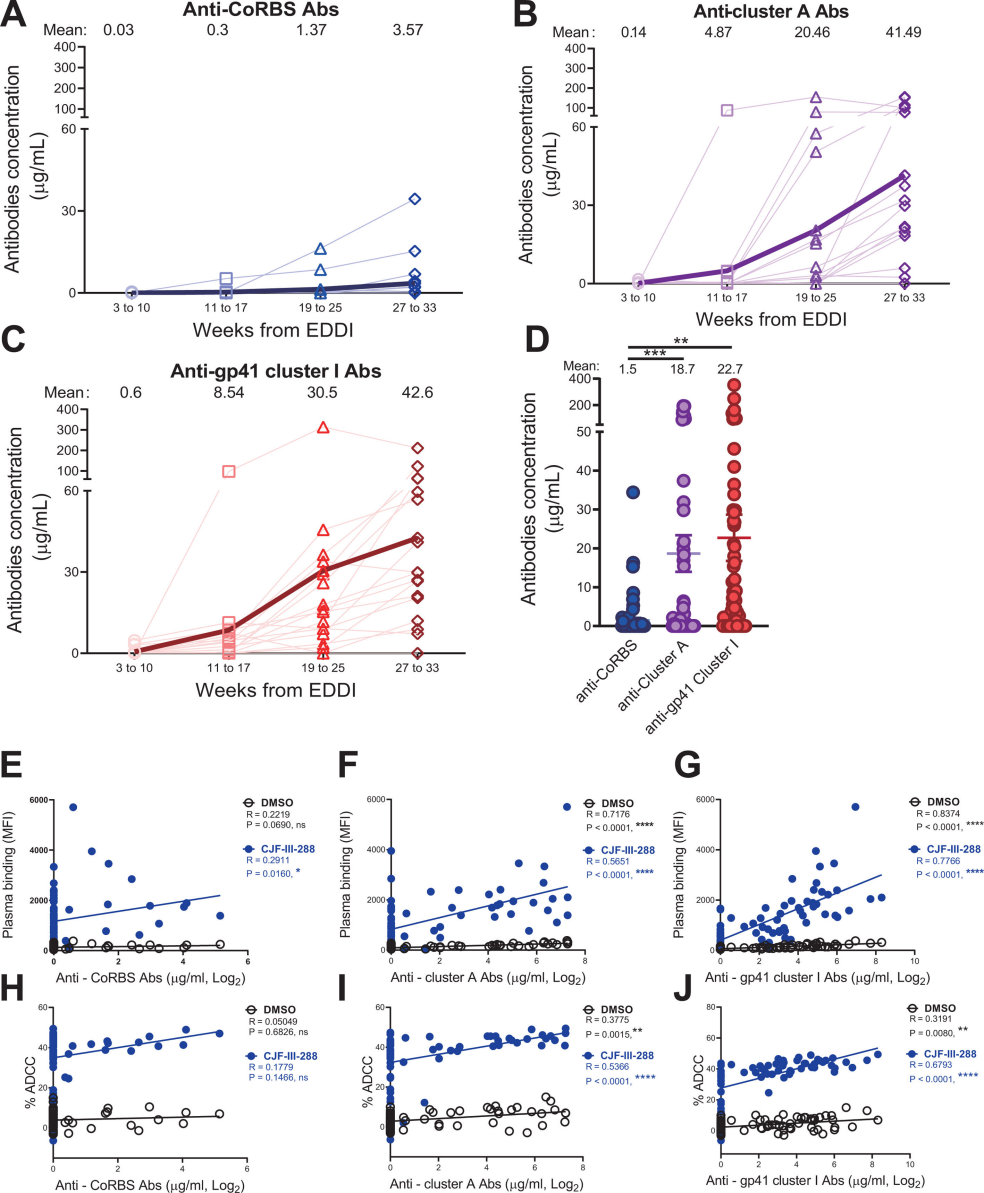
Kinetics of CD4i nnAbs detection in plasma from PLWH. Indirect ELISA was performed with plasma samples from PLWH at different weeks from EDDI to measure the levels of different families of Ab: the anti-CoRBS Abs, blue (**A**), anti-cluster A Abs, purple (**B**), and anti-gp41 cluster I Abs, red (**C**) (see Materials and Methods for more details). For each family of Ab, the mean of the concentration of tested plasma samples from 4 visits of 17 participants is shown (**D**). Spearman correlations between staining (MFI) (E–G) or ADCC (H–J) in the presence of 5 µM of CJF-III-288 (blue) or an equivalent volume of DMSO (black) to the level of anti-CoRBS Abs (**E, H**), anti-cluster A Abs (**F, I**), or anti-gp41 cluster I Abs (**G, J**) were calculated (*P < 0.05; **P < 0.01; ***P < 0.001; ****P < 0.0001). For Fig. D, statistical significance was tested by one-way analysis of variance (ANOVA) (**P < 0.01; ***P < 0.001).

To better understand how the different families of CD4i Abs appear in the plasma from PLWH, we adapted an enzyme-linked immunosorbent assay (ELISA) to quantify CD4i non-neutralizing antibodies (nnAbs). Previous work (20) has shown that three families of CD4i nnAbs (anti-CoRBS, anti-cluster A, and anti-gp41 cluster I Abs) contribute to the ability of plasma from PLWH to mediate ADCC in the presence of CD4mc. Here, we also measured the amount of anti-CoRBS Abs (17b-like) (Fig. 2A), anti-cluster A Abs (A32-like) (Fig. 2B), and anti-gp41 cluster I Abs (F240-like) (Fig. 2C) in plasma. We performed an ELISA assay where plates were coated with a gp120 core (gp120_core_ ΔV1V2V3V5) that spontaneously samples the CD4-bound conformation (34), therefore readily exposing gp120 CD4i epitopes, in the presence or absence of a 17b Fab fragment (which blocks binding of 17b-like anti-CoRBS Abs). We also used the gp120 inner domain (ID2) probe that efficiently exposes the A32 epitope (35, 36) or a gp41 C-C loop peptide (residues 583–618 of the gp41 cluster I region) (37). Both anti-cluster A and anti-gp41 cluster I Abs were detected in the plasma from week 3 to 10 post-EDDI (Fig. 2B and C), while a low level of anti-CoRBS Abs was detected only after 11 weeks post-EDDI (Fig. 2A). Overall, the levels of anti-CoRBS Abs were approximately 10 times lower than the anti-cluster A or anti-gp41 cluster I Abs (Fig. 2D). In the presence of CJF-III-288, significant correlations were observed between staining (Fig. 2E through 2G), ADCC (Fig. 2H through 2J), and the amount of anti-cluster A Ab (Fig. 2F and I) or anti-gp41 cluster I Ab (Fig. 2G and J). No significant correlation was observed for anti-CoRBS Abs (Fig. 2E and H), which could be due to the low levels of anti-CoRBS Abs detected. Altogether, we observed different kinetics of nnAbs elicitation, with anti-cluster A Abs and anti-gp41 cluster I Abs appearing early (3–10 weeks from EDDI), and anti-CoRBS Abs emerging later (after 11 weeks).

### ADCC activity of plasma from PLWH increased over time with the appearance of anti-CoRBS Abs

**Fig 3:**
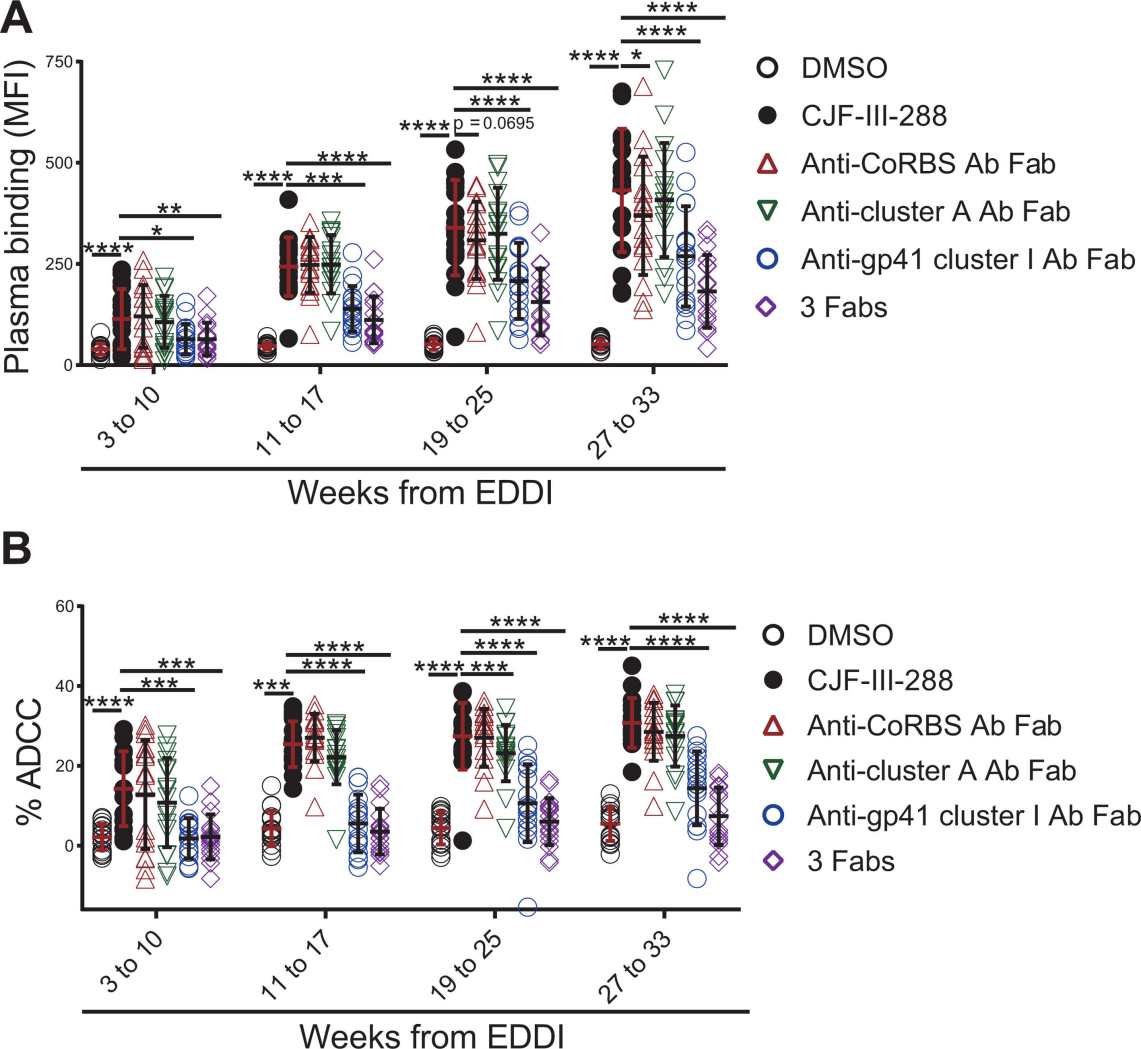
Three CD4i families of antibodies contribute to recognition of HIV-1-infected cells and ADCC in the presence of the CJF-III-288 CD4mc. Fabs competition for Env staining (**A**) and ADCC (**B**) were performed with HIV-1_CH58T/F_ infected primary CD4+ T cells and 1:1,000 diluted plasma samples from PLWH separated according to the weeks from EDDI. Infected cells were pre-incubated with 17b Fab (anti-CoRBS Fab, red triangle), A32 Fab (anti-cluster A Fab, green down-pointing triangle), 246D Fab (F240-like anti-gp41 cluster I Fab, blue circle) or the three Fab fragments together (purple diamond) in the presence of 5 µM of CJF-III-288 or an equivalent volume of DMSO (black circle). Error bars indicate SEM. Statistical significance was tested using one-way ANOVA (parametric) or Friedman test (non-parametric) (*P < 0.05; **P < 0.01; ***P < 0.001; ****P < 0.0001).

To measure the contribution of the three different families of CD4i antibodies to CD4mc sensitization of infected cells to ADCC, we performed a Fab competition experiment. Briefly, HIV-1_CH58T/F_ infected cells were pre-incubated with CJF-III-288 in the presence of 17b Fab (anti-CoRBS Fab), A32 Fab (anti-cluster A Fab), 246D Fab (F240-like anti-gp41 cluster I Fab), or an equimolar combination of the three Fab fragments. Binding of Fab fragments to their cognate epitopes on cell-surface Env prevents the binding of the same family of Abs to infected cells, therefore blocking ADCC, as previously described (20, 28). The Fab fragments of the three families of Abs competed with the Abs present in plasma from all groups and decreased both the recognition of infected cells and ADCC (Fig. 3). However, in the plasma from early infected PLWH (weeks 3 to 10 post-EDDI), the effect of anti-CoRBS Abs was weak, with anti-gp41 cluster I Abs contributing the most to recognition of infected cells and ADCC (Fig. 3, blue circles). With the detection of anti-CoRBS Abs at later time points (Fig. 2A), staining and ADCC increased (Fig. 3, red triangle). Overall, in plasma from early infected PLWH (before 10 weeks after EDDI), anti-gp41 cluster I Abs had the predominant role in recognizing HIV-1 Env and inducing ADCC in the presence of the CD4mc—CJF-III-288.

### CD4mc sensitizes HIV-1 to neutralization by non-neutralizing Abs in plasma from early infected PLWH

**Fig 4:**
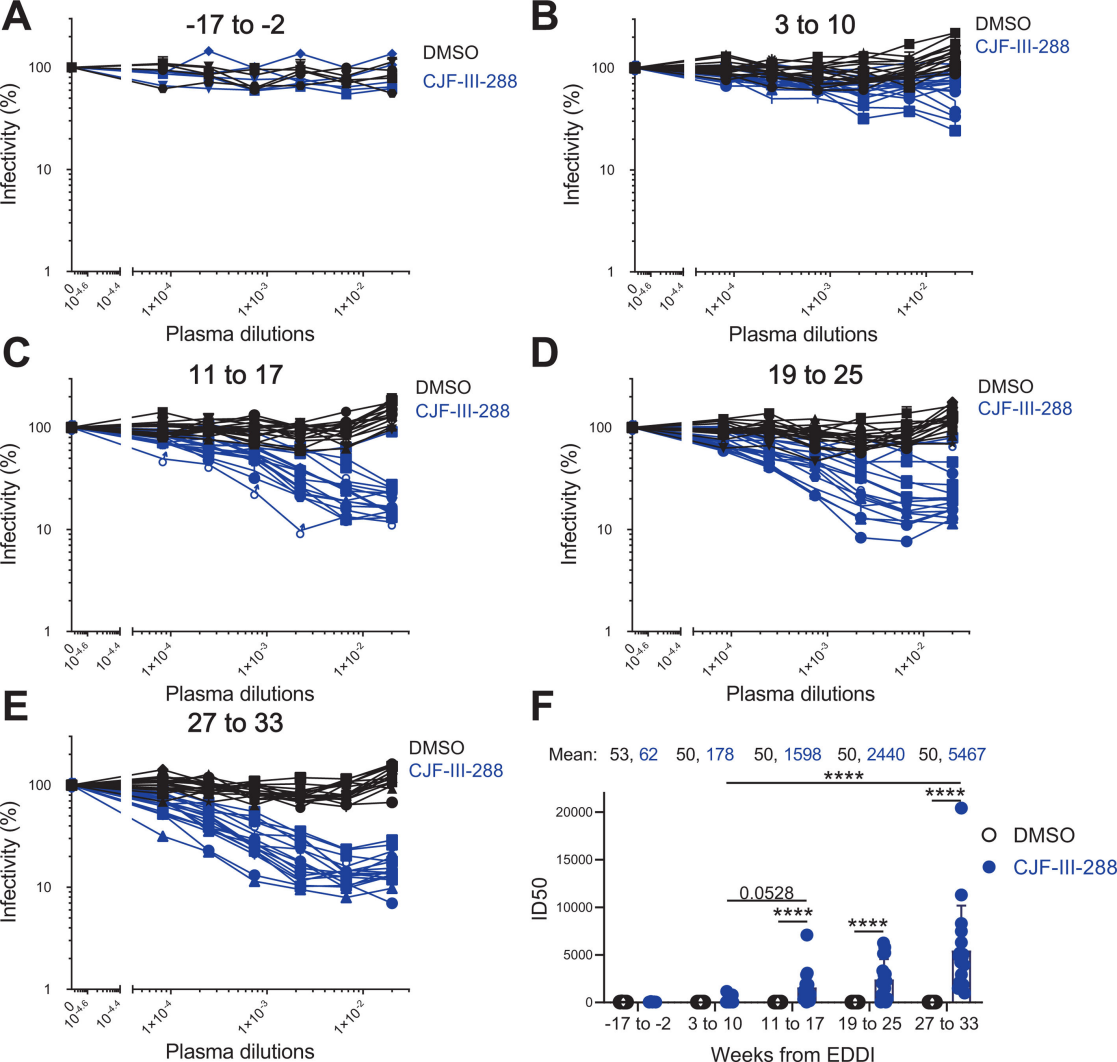
Addition of CD4mc sensitizes virus to neutralization by non-neutralizing Abs in plasma from early infected PLWH. HIV-1_CH58T/F_ virus was pre-incubated with 0.024 µM of CJF-III-288 (which is the IC_50_ value of CJF-III-288 against HIV-1_CH58T/F_ [Fig. S1]) (A to F, blue) or an equivalent volume of DMSO (A to F, black) for 1 h before the incubation with serial diluted (start from 1/50) plasma samples from different groups according to the weeks post-EDDI: −17 to −2 (**A**), 3 to 10 (**B**), 11 to 17 (**C**), 19 to 25 (**D**), and 27 to 33 (**E**). After infection, luciferase activity (RLU) in the cells was measured, and relative infectivity (%) was determined by the ratio of every value to the condition in the absence of plasma. ID_50_ for each plasma was calculated with GraphPad Prism and plotted (ID_50_ that is less than 50, which is lower than the limit of plasma dilution, is considered as 50) (**F**). For each group of samples, the mean values of ID_50_ in the presence of CJF-III-288 or DMSO were shown on the top (**F**). Statistical significance was tested using the Kruskal-Wallis test (*P < 0.05; **P < 0.01; ***P < 0.001; ****P < 0.0001).

It is well-known that CD4mcs sensitize HIV-1 to neutralization by otherwise non-neutralizing antibodies present in plasma from PLWH, as well as plasma from vaccinated humans and non-human primates immunized with Env-based immunogens (22, 38, 39). We tested the neutralization capacity of the plasma samples from early infected PLWH in the presence of a sub-neutralizing concentration of CD4mc—CJF-III-288. We first determined the IC_50_ value (0.024 µM) of CJF-III-288 against HIV-1_CH58T/F_ (Fig. S1). We then evaluated if viral particles pre-incubated with this concentration of CJF-III-288 became susceptible to neutralization by plasma from early stages of HIV-1 infection (Fig. 4, blue). As a control, an equivalent volume of the vehicle dimethyl sulfoxide (DMSO) was added (Fig. 4, black). Both control and compound-virus mixtures were subsequently incubated with the indicated dilutions of plasma in the five groups of samples collected from PLWH according to the time post-EDDI (Table 2 and Fig. 4). Luciferase activity was measured 48 h after infection in TZM-bl cells, and the relative infectivity (%) was calculated by the ratio of every value to the condition in the absence of plasma (Fig. 4A through 4E). The ID_50_ of each plasma sample in the presence of DMSO or CJF-III-288 was calculated and presented in Fig. 4F. As expected, plasma samples collected before HIV-1 infection (weeks from EDDI: −17 to −2) did not neutralize viral particles in the presence or absence of CJF-III-288 (Fig. 4A). None of the samples neutralized viral particles in the absence of CD4mc. The addition of CJF-III-288 enabled neutralization by plasma samples collected as early as 3 to 10 weeks post-EDDI. The neutralization capacity, in the presence of CJF-III-288, increased concomitantly with time post-EDDI (Fig. 4C through 4E, blue), with a mean ID_50_ of 1,598 for group “11 to 17,” 2,440 for group “19 to 25,” and 5,467 for group “27 to 33” (Fig. 4F). Altogether, these results show that antibodies elicited in the early stages of HIV-1 infection have the capacity to neutralize viral particles in the presence of CD4mc.

## DISCUSSION

HIV-1 infection establishes a reservoir in the first weeks of infection (1). ART treatment controls viral replication but does not eradicate the viral reservoir (2). Our study with plasma from people with early-stage HIV confirmed that Abs in plasma collected in weeks 3 to 10 post-EDDI have the capacity to neutralize viral particles and eliminate HIV-1-infected cells by ADCC in the presence of a CD4mc.

We were able to analyze the kinetics of antibody production and ADCC due to the unique sample set provided through the Sabes study. Unlike studies that identify participants with acute infection through cross-sectional screening (as HIV RNA positive/HIV antibody negative), the Sabes study identified incident HIV infections via frequent HIV testing (RNA and serology) of closely monitored participants at high risk of HIV acquisition. Participants newly diagnosed with HIV infection were rapidly linked to the study clinical site, enrolled, and followed with frequent assessments and specimen collection. While this approach is expensive and time-consuming, it offers many advantages, including the following: (i) prompt identification of HIV acquisition allowing sample collection for targeted studies of acute/early HIV, (ii) detailed testing histories which allowed a more accurate estimation of the time of HIV acquisition, (iii) collection of pre-infection samples which can be used as individual-level controls in laboratory analysis, and (iv) an unbiased assessment of early clinical events such as acute retroviral syndrome.

It has been reported on several occasions that CD4mc sensitizes HIV-1-infected cells to ADCC mediated by non-neutralizing antibodies (20, 23, 25–28, 40). In this study, we observed that nnAbs belonging to three families of CD4i Abs, anti-CoRBS, anti-cluster A, and anti-gp41 cluster I, are elicited early after HIV-1 acquisition, with anti-gp41 cluster I Abs being detected as early as 3 weeks post-EDDI. These three families of nnAbs were recently reported to contribute to the elimination of HIV-1-infected cells by ADCC in the presence of CD4mcs (20, 28). CD4mc engagement in the Phe43 cavity induces conformational changes in Env, which result in the exposure of the coreceptor binding site (21, 22). Anti-CoRBS Ab binding to Env induces additional downstream conformational changes, exposing the gp120 inner domain cluster A region (41, 42). The Fc region of these Abs recruits Fcγ receptor-expressing cells in peripheral blood mononuclear cells (PBMCs) to mediate ADCC (43). Using longitudinal plasma samples from Sabes study participants with early HIV infection, we were able to show that recognition of HIV-1-infected cells and ADCC progressively increased over time, reaching a plateau of ADCC by 19 to 25 weeks after EDDI (Fig. 2A and B). ADCC activity increased concomitantly with the elicitation of CD4i Abs and improved over time with the appearance of anti-CoRBS Abs. This supports previous results showing that there is a sequential opening of the trimer initiated by CD4mc. CD4mc provides an initial opening of the trimer; anti-CoRBS Abs contribute to additional conformational changes required to expose the gp120 inner domain layers 1 and 2. Thus, enabling exposure of the otherwise occluded cluster A region, which is recognized by anti-cluster A antibodies. These antibodies were shown to contribute to plasma-mediated ADCC in the presence of CD4mc (41–44).

Finally, our results show that CD4i nnAbs, able to eliminate HIV-1-infected cells, are elicited within a few weeks after HIV-1 acquisition and suggest that CD4mcs might be used as an early intervention to kill HIV-infected cells and thereby decrease the size of the viral reservoir.

## MATERIALS AND METHODS

Materials and methods have been previously reported in references 12, 23–26, 35–37, 40, 45–51 and are summarized below.

### Small CD4-mimetic

CJF-III-288 was prepared as previously described (25). The compound was determined to be >95% pure based on ^1^H NMR.

### Virus production

HIV-1 viruses for staining and ADCC were produced as previously described (12, 24). Briefly, plasmid encoding the full-length infectious molecular clone (IMC) of HIV-1_CH58T/F_ or HIV-1_CH77T/F_ and plasmid encoding vesicular stomatitis virus G were co-transfected in 293T cells using polycation polyethylenimine (PEI). Two days later, cell supernatants were harvested, clarified by low-speed centrifugation (5 min at 1,500 rpm), and concentrated by ultracentrifugation for 1 h at 4°C at 143,260 × *g* over a 20% sucrose cushion. Pellets were harvested in fresh RPMI media, and aliquots were stored at −80°C until use. Viruses were then used to infect activated primary CD4+ T cells from healthy HIV-uninfected donors via spin infection at 800 × *g* for 1 h in 96-well plates at 25°C; 48 h later, ~15% of cells were infected as measured by intracellular p24 staining. For the virus production for neutralization, a plasmid coding the full-length IMC of HIV-1_CH58T/F_ was transfected in 293T cells using PEI. Two days later, cell supernatants were harvested, clarified by low-speed centrifugation (5 min at 1,500 rpm), and stored at −80°C until use.

### Antibody production and purification

FreeStyle 293F cells (Thermo Fisher Scientific) were grown in FreeStyle 293F medium (Thermo Fisher Scientific) to a density of 1 × 10^6^ cells/mL at 37°C with 8% CO_2_ with regular agitation (150 rpm). Cells were transfected with plasmids expressing the light and heavy chains of the anti-cluster A A32, the anti-CoRBS 17b, or the anti-gp41 cluster I F240 and 246D antibodies using ExpiFectamine 293 transfection reagent, as directed by the manufacturer (Thermo Fisher Scientific). One week later, the cells were pelleted and discarded. The supernatants were filtered (0.22-μm-pore-size filter), and antibodies were purified by protein A affinity columns, as directed by the manufacturer (Cytiva, Marlborough, MA, USA). The recombinant protein preparations were dialyzed against phosphate-buffered saline (PBS) and stored in aliquots at −80°C. To assess purity, recombinant proteins were loaded on SDS-PAGE polyacrylamide gels in the presence or absence of β-mercaptoethanol and stained with Coomassie blue. The A32, 17b, and 246D Fab fragments were prepared from purified IgG (10 mg/mL) by proteolytic digestion with immobilized papain at 37°C (Pierce, Rockford, IL) and purified using protein A, followed by gel filtration chromatography on a Superose 6 10/300 column (Cytiva).

### Protein production

Gp120 inner domain (ID2) was expressed and isolated as previously described (36). Briefly, stable HEK293 cell lines containing the ID2 expression plasmid were cultured for 6–7 days before the collection of the supernatant and passage through a 0.22 µm filter. Medium was then run over an N5-i5 affinity column (a protein A column with crosslinked N5-i5 IgG; N5-i5 is a cluster A Ab), washed thoroughly with PBS, and eluted with 0.1 M glycine at pH 3. ID2 was analyzed via SDS-PAGE, dialyzed into PBS, and sterile filtered.

For gp120 core ΔV1V2V3V5 production, FreeStyle 293F cells (Invitrogen, Carlsbad, CA, USA) were grown in FreeStyle 293F medium (Invitrogen) to a density of 1 × 10^6^ cells/mL at 37°C with 8% CO_2_ with regular agitation (125 rpm). Cells were transfected with a pCDNA3.1 plasmid encoding codon-optimized His(6)-tagged HIV-1_YU2_ gp120 core ΔV1V2V3V5 using the ExpiFectamine 293 Transfection Kit as directed by the manufacturer (Invitrogen). One week later, cells were pelleted and discarded. The supernatants were filtered (0.22-μm-pore-size filter) (ThermoFisher Waltham, MA, USA), and the gp120 glycoproteins were purified by nickel affinity columns according to the manufacturer’s instructions (Invitrogen). Fractions containing gp120 were concentrated using Centriprep-30K (EMD Millipore Billerica, MA, USA) centrifugal filter units following the manufacturer’s instructions. Monomeric gp120 was then purified by gel filtration chromatography as described (50). Monomeric gp120 preparations were dialyzed against PBS buffer and stored in aliquots at −80°C. To assess purity, recombinant proteins were loaded on SDS-PAGE gels and stained with Coomassie blue.

The gp41 peptide synthesis was previously described (37). The amino acid sequence of the 36-residue peptide of the sequence 583–618 of gp41 (based on the clade B BaL sequence) is gp41 583–618: VERYLRDQQLLGIWGCSGKLICTTAVPWNASWSNKS. The peptide was synthesized on an ABI 433A automated peptide synthesizer using the optimized HBTU activation/DIEA *in situ* neutralization protocol developed by Kent and colleagues for Boc-chemistry solid phase peptide synthesis. After cleavage and deprotection in HF, the crude product was precipitated with cold ether and purified to homogeneity by preparative C18 reversed-phase high-performance liquid chromatography (RP-HPLC) to afford reduced peptide. The molecular masses were confirmed by electrospray ionization mass spectrometry (ESI-MS). Mass: observed 4,082.0 Da, calculated 4,081.6 Da. The reduced peptide was dissolved at 0.4 mg/mL in 1 M GuHCl containing 20% DMSO (vol/vol) for disulfide formation. After 2 h, the reaction was completed and purified with RP-HPLC to afford the oxidized peptide. Mass (ESI): observed 4,079.9 Da, calculated 4,079.6 Da.

### Flow cytometry analysis of cell-surface staining

Cell-surface staining was performed as previously described (23, 26, 45). Primary CD4+ T cells were isolated from healthy HIV-uninfected donors and infected with HIV-1_CH58T/F_ or HIV-1_CH77T/F_. Binding of HIV-1-infected cells with plasma (1:1,000 dilution) in the presence or absence of a potent CD4mc, CJF-III-288 (5 µM), was performed 48 h after infection. Of note, all plasma samples were tested in parallel using cells from a single individual, one for HIV-1_CH58T/F_ or HIV-1_CH77T/F_. PBMCs from a third individual were used for the Fab blockade experiments. Briefly, HIV-1-infected cells were pre-incubated for 15 min at room temperature with DMSO/CJF-III-288 and 17b Fab (20 µg/mL), A32 Fab (20 µg/mL), 246D Fab (40 µg/mL), or the three Fab fragments together before addition of PLWH plasma. Cells were then incubated at 37°C for 1 h followed by adding Alexa Fluor 647 conjugated anti-human IgG (Invitrogen, Waltham, MA, USA) secondary Ab or Alexa-Fluor-647-conjugated anti-human IgG Fc secondary antibodies (for Fab blockade experiments) for 20 min and FITC labeled anti-human CD4 Ab (OKT-4-FITC) (Biolegend, San Diego, CA, USA) for another 20 min. Cells were then stained intracellularly for HIV-1 p24, using the Cytofix/Cytoperm Fixation/Permeabilization Kit (BD Biosciences, Mississauga, ON, Canada) and the fluorescent anti-p24 mAb (PE-conjugated anti-p24, clone KC57; Beckman Coulter/Immunotech). The percentage of infected cells (CD4low/p24+ cells) was determined by gating the live cell population on the basis of the AquaVivid viability dye (Thermo Fisher Scientific, Waltham, MA, USA). Samples were analyzed on an LSRII cytometer (BD Biosciences, Bad Wildbad, Germany), and data analysis was performed using FlowJo 10.10.0 (Tree Star, Ashland, OR, USA).

### ADCC assay

ADCC was performed with a FACS-based assay as previously described (46–48). Briefly, HIV-1_CH58T/F_ or HIV-1_CH77T/F_ infected primary CD4+ T cells were stained with the viability dye AquaVivid (Thermo Fisher Scientific, Waltham, MA, USA) and the cellular proliferation eFluor670 marker (eBioscience, San Diego, CA, USA) and were used as target cells. NK cell line KHYG-1 cells (52) stained with another cellular marker—cell proliferation dye eFluor450 (eBioscience, San Diego, CA, USA) were added at an effector/target ratio of 10:1 in 96-well V-bottom plates (Corning, Corning, NY, USA). Then, the mixed cells were incubated with plasma from PLWH (1:1,000), in the presence of 5 µM CJF-III-288 or with an equivalent volume of vehicle (DMSO). For blockade experiments, cells were pre-incubated for 15 min at room temperature with DMSO/CJF-III-288 and 17b Fab (20 µg/mL), A32 Fab (20 µg/mL), 246D Fab (40 µg/mL), or the three Fabs before the addition of plasma and effector cells. The plates were subsequently centrifuged for 1 min at 300 × *g* and incubated at 37°C, 5% CO_2_ for 4 to 6 h; FITC-labeled anti-human CD4 Ab (OKT-4-FITC) (Biolegend, San Diego, CA, USA) was used for CD4 staining for 20 min. Cells were then stained intracellularly for HIV-1 p24, using the Cytofix/Cytoperm Fixation/Permeabilization Kit (BD Biosciences, Mississauga, ON, Canada) and the fluorescent anti-p24 mAb (PE-conjugated anti-p24, clone KC57; Beckman Coulter/Immunotech). Samples were analyzed on an LSRII cytometer (BD Biosciences, Bad Wildbad, Germany). The percentage of infected cells (CD4low/p24+ cells) was determined by gating the live cell population on the basis of the AquaVivid viability dye (Thermo Fisher Scientific, Waltham, MA, USA). Data analysis was performed using FlowJo vX.0.7 (Tree Star, Ashland, OR, USA). The percentage of ADCC was calculated with the following formula: (% of CD4low/p24+ cells in Targets plus Effectors) − (% of CD4low/p24+ cells in Targets plus Effectors plus plasma)/(% of CD4low/p24+ cells in Targets).

### Anti-cluster A ELISA

Recombinant stabilized gp120 inner domain (ID2) exposing the A32 epitope was previously described (35). The recombinant proteins were prepared in PBS at a concentration of 0.1 µg/mL and were adsorbed to white 96-well plates (MaxiSorp Nunc) overnight at 4°C. Coated wells were subsequently blocked with blocking buffer (Tris-buffered saline [TBS] containing 0.1% Tween 20 and 2% bovine serum albumin [BSA]) for 1 h at room temperature. Wells were then washed four times with washing buffer (TBS containing 0.1% Tween 20). Plasma samples (dilution 1:8,000), twofold serial dilution of anti-cluster A A32 Ab (from 50 to 0.4 ng/mL) to determine concentration using a standard curve, and another dilution of A32 at 1 µg/mL for normalization between plates was prepared in a diluted solution of blocking buffer (0.1% BSA in TBS) and incubated with coated wells for 90 min at room temperature. Plates were washed four times with washing buffer followed by incubation with horseradish peroxidase (HRP)-conjugated anti-human IgG secondary Ab (Cat #:31410, Invitrogen. 0.3 µg/mL in a diluted solution of blocking buffer [0.4% BSA]) for 1 h at room temperature, followed by four washes. HRP enzyme activity was determined after the addition of a 1:1 mix of Western Lightning Plus-ECL oxidizing and luminol reagents (PerkinElmer Life Sciences). Light emission was measured with an LB942 TriStar luminometer (Berthold Technologies). The signal obtained with BSA was subtracted from each plasma and normalized to the signal obtained with A32 present in each plate. The concentration of anti-cluster A Abs in plasma was determined with the A32 standard curve. The seropositivity threshold was established using the following formula: mean of 4 plasma samples from HIV-1-uninfected individuals + (3 × standard deviation of the mean of 4 HIV-negative plasma).

### Anti-CoRBS ELISA

Recombinant CD4-bound stabilized gp120 proteins, lacking V1, V2, V3, and V5 regions (gp120 core ΔV1V2V3V5) were previously described (49, 50). Proteins were prepared in PBS at a concentration of 0.1 µg/mL and were adsorbed to white 96-well plates (MaxiSorp Nunc) overnight at 4°C. Coated wells were subsequently blocked with blocking buffer (TBS containing 0.1% Tween 20 and 2% BSA) for 1 h at room temperature. Wells were then washed four times with washing buffer (TBS containing 0.1% Tween 20). Anti-CoRBS 17b Fab fragment (0.5 µg/mL) was prepared in a diluted solution of blocking buffer (0.1% BSA). The other half of the plate was incubated with a solution of blocking buffer (0.1% BSA). Wells were then washed four times with washing buffer (TBS containing 0.1% Tween 20). Plasma samples (dilution 1:8,000), twofold serial dilution of anti-CoRBS 17b Ab (from 50 to 0.4 ng/mL) to determine concentration using a standard curve, and another dilution of 17b at 1 µg/mL for normalization between plates was prepared in a diluted solution of blocking buffer (0.1% BSA) and incubated with the peptide-coated wells for 90 min at room temperature. Plates were washed four times with washing buffer, followed by incubation with HRP-conjugated anti-human IgG secondary Ab (Cat #:31410, Invitrogen, 0.3 µg/mL in a diluted solution of blocking buffer [0.4% BSA]) for 1 h at room temperature, followed by four washes. HRP enzyme activity was determined after the addition of a 1:1 mix of Western Lightning Plus-ECL oxidizing and luminol reagents (PerkinElmer Life Sciences). Light emission was measured with an LB942 TriStar luminometer (Berthold Technologies). The signal obtained with BSA was subtracted from each plasma and was then normalized to the signal obtained with 17b present in each plate. Concentration of the plasma was determined with the 17b standard curve. The anti-CoRBS antibody level corresponds to the value obtained when the concentration obtained with 17b Fab preincubation condition is subtracted from that without 17b Fab preincubation condition. The seropositivity threshold was established using the following formula: mean of 4 plasma samples from HIV-1-uninfected individuals + (3 × standard deviation of the mean of 4 HIV-negative plasma).

### Anti-gp41 cluster I peptide ELISA

Peptides corresponding to the gp41 C-C loop region (residues 583–618) were either previously described (37, 40) or purchased from Genscript (Piscataway, NJ, USA). Peptides were prepared in PBS at a concentration of 0.1 µg/mL and were adsorbed to white 96-well plates (MaxiSorp Nunc) overnight at 4°C. Coated wells were subsequently blocked with blocking buffer (TBS containing 0.1% Tween 20 and 2% BSA) for 1 h at room temperature. Wells were then washed four times with washing buffer (TBS containing 0.1% Tween 20). Plasma samples (dilution 1:8,000), twofold serial dilution of anti-gp41 cluster I F240 Ab (from 50 to 0.4 ng/mL) to determine concentration using a standard curve, and another dilution of F240 at 1 µg/mL for normalization between plates was prepared in a diluted solution of blocking buffer (0.1% BSA) and incubated with the peptide-coated wells for 90 min at room temperature. Plates were washed four times with washing buffer, followed by incubation with HRP-conjugated anti-human IgG secondary Ab (Cat #:31410, Invitrogen, 0.3 µg/mL in a diluted solution of blocking buffer [0.4% BSA]) for 1 h at room temperature, followed by four washes. HRP enzyme activity was determined after the addition of a 1:1 mix of Western Lightning Plus-ECL oxidizing and luminol reagents (PerkinElmer Life Sciences). Light emission was measured with an LB942 TriStar luminometer (Berthold Technologies). The signal obtained with BSA was subtracted from each plasma and was then normalized to the signal obtained with F240 present in each plate. The concentration of the plasma was determined with the F240 standard curve. The seropositivity threshold was established using the following formula: mean of 4 plasma samples from HIV-1-uninfected individuals + (3 × standard deviation of the mean of 4 HIV-negative plasma).

### Virus neutralization

Virus neutralization was performed as previously described (51). Twenty-four hours before infection, TZM-bl cells were seeded at a density of 5 × 10^4^ cells/well in 96-well luminometer-compatible tissue culture white plates (Perkin Elmer). For IC_50_ measurement of CJF-III-288 against HIV-1_CH58T/F_, 3× serial dilutions of CJF-III-288 starting from 2 µM were incubated with the virus for 1 h at 37°C, and the mixture was added to the cells for infection for at least 48 h. For ID_50_ measurement of tested plasma samples, HIV-1_CH58T/F_ was first incubated with 0.024 µM of CJF-III-288 (IC_50_ of CJF-III-288 against HIV-1_CH58T/F_) or an equivalent volume of DMSO for 1 h at 37°C. Then, 3× serial dilutions of tested plasma samples starting from 1/50 were added to the virus and incubated for another 1 h at 37°C. Finally, the mixture was added to the cells for infection for at least 48 h. Then the cells were lysed by the addition of 30 µL of passive lysis buffer (Promega) and followed by three freeze-thaw cycles. After the addition of 100 µL of luciferin buffer (15 mM MgSO_4_, 15 mM KH_2_PO_4_ [pH 7.8], 1 mM ATP, and 1 mM dithiothreitol) and 50 µL of 1 mM D-luciferin potassium salt (Prolume), an LB941 TriStar luminometer (Berthold Technologies) was used to measure the luciferase activity. The neutralization half-maximal inhibitory concentration (IC_50_) represents the concentration of the tested compound to inhibit 50% of the virus infection of target cells, and the neutralization half-maximal inhibitory dilution (ID_50_) represents the dilution of plasma to inhibit 50% of the virus infection of target cells.

### Quantification and statistical analysis

Statistics were analyzed using GraphPad Prism version 8.4 (GraphPad, San Diego, CA, USA). Every data set was tested for statistical normality, and this information was used to apply the appropriate (parametric or non-parametric) statistical test. Statistical details of experiments are indicated in the figure legends. *P* values <0.05 were considered significant; significance values are indicated as *P < 0.05, **P < 0.01, ***P < 0.001, ****P < 0.0001.

## Data Availability

Data and reagents are available upon request.

## SUPPLEMENTAL MATERIAL

Figure S1 jvi.00858-25-s0001.pdf Measurement of IC_50_ value of CJF-III-288 against HIV-1_CH58T/F_.

ASM does not own the copyrights to Supplemental Material that may be linked to, or accessed through, an article. The authors have granted ASM a non-exclusive, world-wide license to publish the Supplemental Material files. Please contact the corresponding author directly for reuse.
