# Supplementary material for: CD4-mimetics sensitize HIV-infected cells to ADCC mediated by plasma from persons with early-stage HIV-1 infection
Source: J Virol. 2025 Jul 21;99(8):e00858-25. doi: 10.1128/jvi.00858-25 (PMC12363219; doi:10.1128/jvi.00858-25)
Supplement: Figure S1 — Measurement of IC50 value of CJF-III-288 against HIV-1CH58T/F. [file jvi.00858-25-s0001.pdf]

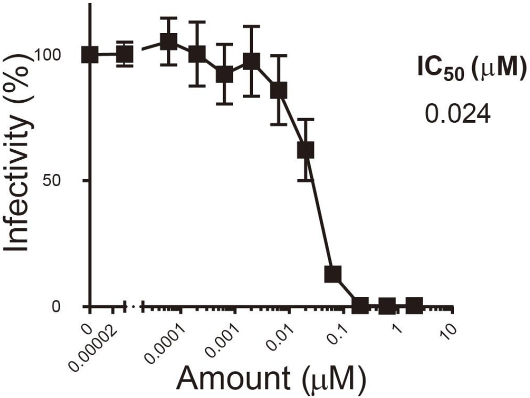

Figure S1. Measurement of IC_{50} value of CJF-III-288 against HIV-$1_{CH58T/F}$. HIV-$1_{CH58T/F}$ virus was incubated with indicated serial diluted concentrations (start from 2 μM) of CJF-III-288 for 1 hour before the infection in TZM-bl cells. 48 hours after infection, luciferase activity (RLU) in the cells was measured and relative infectivity (%) was determined by the ratio to that in the absence of CJF-III-288. IC_{50} was calculated with GraphPad Prism and shown.
